# Differential expression profiling between the relative normal and dystrophic muscle tissues from the same LGMD patient

**DOI:** 10.1186/1479-5876-4-53

**Published:** 2006-12-19

**Authors:** Yong Zhang, Jianwei Ye, Dazhi Chen, Xinyi Zhao, Xingjun Xiao, Sheng Tai, Wei Yang, Dahai Zhu

**Affiliations:** 1 National Laboratory of Medical Molecular Biology, Institute of Basic Medical Sciences, Chinese Academy of Medical Sciences and Peking Union Medical School, Beijing, 100005, P. R. China; 2 Department of General Surgery, Chaoyang Hospital, Capital Medical University, Beijing, 100020, P. R. China; 3 Molecular and Cellular Developmental Biology Laboratory, Harbin Institute of Technology, Harbin, 150001, P. R. China; 4 Department of Neurology, Second Affiliated Hospital, Harbin Medical University, Harbin, 150086, P. R. China; 5 Department of General Surgery, Second Affiliated Hospital, Harbin Medical University, Harbin, 150086, P. R. China

## Abstract

**Background:**

Limb-girdle muscular dystrophy (LGMD) is a group of heterogeneous muscular disorders with autosomal dominant and recessive inheritance, in which the pelvic or shoulder girdle musculature is predominantly or primarily involved. Although analysis of the defective proteins has shed some light onto their functions implicated in the etiology of LGMD, our understanding of the molecular mechanisms underlying muscular dystrophy remains incomplete.

**Methods:**

To give insight into the molecular mechanisms of AR-LGMD, we have examined the differentially expressed gene profiling between the relative normal and pathological skeletal muscles from the same AR-LGMD patient with the differential display RT-PCR approach. The research subjects came from a Chinese AR-LGMD family with three affected sisters.

**Results:**

In this report, we have identified 31 known genes and 12 unknown ESTs, which were differentially expressed between the relative normal and dystrophic muscle from the same LGMD patient. The expression of many genes encoding structural proteins of skeletal muscle fibers (such as titin, myosin heavy and light chains, and nebulin) were dramatically down-regulated in dystrophic muscles compared to the relative normal muscles. The genes, reticulocalbin 1, kinectin 1, fatty acid desaturase 1, insulin-like growth factor binding protein 5 (IGFBP5), Nedd4 family interacting protein 1 (NDFIP1), SMARCA2 (SWI/SNF related, matrix associated, actin dependent regulator of chromatin, subfamily a, member 2), encoding the proteins involved in signal transduction and gene expression regulation were up-regulated in the dystrophic muscles.

**Conclusion:**

The functional analysis of these expression-altered genes in the pathogenesis of LGMD could provide additional information for understanding possible molecular mechanisms of LGMD development.

## Background

The limb-girdle muscular dystrophies (LGMDs) are a group of clinically and genetically heterogeneous disorders of the skeletal muscle inherited in either autosomal dominant or recessive fashion. LGMDs are characterized clinically by progressive muscle weakness predominantly in the pelvic and shoulder-girdle muscles, serum creatine kinase (SCK) elevation, normal intelligence and great variability, ranging from severe forms with onset in the first decade and rapid progression to milder forms with later onset and a slower course [1,2]. The diagnosis of LGMDs can be excluded by the finding of severely abnormal dystrophin staining on muscle biopsies [3].

Autosomal dominant LGMD, named LGMD1/AD-LGMD, contains at least six entities designated LGMD1A to 1F [4-7]. Gene mutations of myotilin, laminA/C or caveolin3 can lead to LGMD1A-1C. The candidate genes for three other types of LGMD1 have not been identified. There are at least ten distinct entities of autosomal recessive LGMD, designated LGMD2/AR-LGMD (LGMD2A to LGMD2J), and a number of genes and their mutations involved in the pathogenesis of LGMD2 have been well-documented [8]. The identification of the gene mutations and protein products involved in the LGMDs provide an avenue to the molecular diagnosis of LGMDs.

Although analysis of the defective proteins has shed some light onto their functions implicated in the etiology of LGMD, our understanding of the molecular mechanisms underlying muscular dystrophy remains incomplete. We still do not know why the different defective proteins can cause the same clinical manifestations, such as muscle weakness and dystrophy, during the disease development. From our current understanding of LGMD development, it seems most likely that many regulatory genes that participate in muscular pathogenesis have not been identified. Thus, one of the remaining challenges in understanding the molecular basis of LGMDs is to identify those regulatory genes that play very important roles in the LGMD pathogenesis. Toward this end, gene expression profiling alterations in LGMD patients, as compared to the normal person, have been reported [9-11].

To contribute to the identification of additional regulatory genes involved in the muscle pathogenesis of AR-LGMD, differential display reverse transcription polymerase chain reactions (DD-RT-PCR) have been used to compare the gene expression differences between the relative normal and pathological dystrophic skeletal muscles from the same LGMD patient. The muscle samples taken from the same patients in this study could reduce the genetic background resulting from the comparison between two different people (one normal and one patient). We have identified 43 differentially expressed genes (31 known genes and 12 new ESTs) between the normal and dystrophic muscles from the same LGMD patient. Expression analyses of those genes indicated that expression of the genes encoding structural proteins of skeletal muscle fibers were significantly down-regulated in dystrophic muscles compared to the relative normal muscles from the same patients. However, the genes encoding the proteins participating in signal transduction and expression regulation were up-regulated in the dystrophic muscles of the same patients. The further functional analyses of these genes in the pathogenesis of LGMD would be of great interest for the better understanding of the molecular mechanisms of LGMD development.

## Methods

### Patients and clinical diagnosis

The patients were out patients from the Department of Neurology of the Second Hospital of Harbin Medical University, Harbin, China. The AR-LGMD patients were diagnosed according to clinical observations, family history, serum creatine kinase (SCK) levels, electromyography (EMG) features and normal dystrophin and emerin expression. Muscle biopsies (quadriceps and deltoids) were obtained with informed consent following protocols based on the institutions.

### Total RNA isolation and DNase treatment

Total RNA was isolated from muscle biopsies using the Trizol reagent (Invitrogen) according to the manufacturer's instructions. Genomic DNA contamination was removed by DNase I digestion at 37°C for 30~40 min. The DNase-treated RNAs were extracted with phenol/chloroform and precipitated with ethanol.

### Reverse transcription for the first-strand cDNA synthesis

Three reverse transcription reactions, each containing one of the three different anchored primers, were performed for each RNA sample. The DNase treated total RNA (1 μg) was used as the template for each reverse transcription reaction. The reactions were carried out according to the protocols of Advantage RT-for-PCR Kit (Clontech). The anchored primers used in these experiments were synthesized by the Shanghai Sangon Corporation, including B0327: 5'-AAGCTTTTTTTTTTG-3', B0328: 5'-AAGCTTTTTTTTTTA-3', B0329: 5'-AAGCTTTTTTTTTTC-3'.

### mRNA differential display

The mRNA differential display (DD-PCR) was performed with a modification of the procedure described by Liang and Pardee [24]. The double-stranded cDNA was synthesized with combinations of arbitrary primers and anchored primers in the presence of the isotope [α-^33^P] dATP. The arbitrary primers used in this experiment were synthesized by the Shanghai Sangon Corporation, including B0305:5'-GGAACCAATC-3', B0306:5'-AAACTCCGTC-3', B0307:5'-TCGATACAGG-3', B0308:5'-TGGTAAAGGG-3', B0309:5'-TCGGTCATAG-3', B0310:5'-GGTACATTGG-3', B0311:5'-TACCTAAGCG-3', B0312:5'-CTGCTTGATG-3', B0313:5'-GTTTTCGCAG-3', B0314:5'-GATCAAGTCC-3'. The PCR reactions contained 1 μl of reverse transcriptional products, 0.2 μl [α-^33^P] dATP(10 mCi/ml, NEN), ExTaq 1.25U (Takara), 2.0 μmol/L anchored primer, 0.5 μmol/L arbitrary primer and 2.0 μmol/L dNTPs. The PCR was performed under the following conditions: 94°C for 3 min and then 35 cycles of 94°C for 30 s, 40°C for 2 min, and 72°C for 1 min. A final extension was carried out at 72°C for 5 min. The PCR products were separated on 6% denaturing polyacrylamide gels at 60 watt for 6 hours. The gel was dried and DNA bands were visualized by autoradiography at -80°C.

### Re-amplification, cloning and sequencing of differentially expressed cDNA fragments

The differential bands were excised from the gel, and DNA was eluted by boiling the gel in 100 μl of H_2_O for 15 min and then precipitated at -70°C for at least 30 min with 10 μl of 3M NaAc, 5 μl of glycogen (20 mg/ml) and 400 μl of 100% ethanol. Precipitated DNA was washed once with 85% ethanol, dissolved in 10 μl of H_2_O and used as the template to re-amplify the fragment by PCR with the same primer set and conditions used for the above DD-PCR reaction. The gel-purified DNA was ligated to pGEM^®^-T Easy vector system (Promega) and sequenced. The sequences were analyzed with the BLASTN program.

### Real time qRT-PCR

PCR analyses were performed using the ABI Prism 7500 System. Real-time quantification employed the SYBR Green PCR Master Mix (ABI). PCR primers were designed using AB PRISM Primer Express 2.0 software. To avoid amplification of contaminating genomic DNA, one of the two primers was placed at or just outside the exon/exon junction (see Table 3). BLASTN searches have been done against sequence databases to confirm gene specificity of the primer sequences. qRT-PCR was performed in triplicates with standard deviations of threshold cycle (CT) values not exceeding 0.5. After a general reverse transcription reaction, PCR analyses were performed in the 20 μl amplification reactions containing 10 μl of SYBR Green PCR Master Mix, 20 ng cDNA and 0.5 μM of each primer at the following conditions: 95°C for 10 minutes × 1 cycle, 40 cycles at 95°C for 15 seconds and then at 60°C for 1 minute. At the end of the PCR, the results were exported to Microsoft Excel for analysis.

**Table 3:** The differential expressions of 14 genes were confirmed by real time qRT-PCR

**No**	**Gene name**	**Primers for real-time qRT-PCR**	**Fold change**
1	Nebulin-related anchoring protein (NRAP), transcript variant 1	F: 5'-GCCTCAGGCATGCTCAGAAG-3' R: 5'-ACTTTGTAGGAGCCAGGAGGG-3'	-76.92
2	Reticulocalbin1, EF hand calcium binding domain	F: 5'-GAAACCCTGGAGGACATCGA-3' R: 5'-TCTGGCTCAGGGCCATTCT-3'	1.99
3	SWI/SNF related, matrix associated, actin dependent regulator of chromatin, subfamily a, member 2 (SMARCA2), transcript variant 2	F: 5'-CATGCTTCTCTGTCACAACGC-3' R: 5'-TCTGCCGGGCACTCTTAAAC-3'	2.02
4	Fatty acid desaturase 1 (FADS1)	F: 5'-CCTTGTGTGCCAAGCATGG-3' R: 5'-TAGCCAGAGCTGCCCTGACT-3'	45.34
5	Voltage-dependent anion channel 1	F: 5'-CAAAATCCCGAGTGACCCAG-3' R: 5'-GGAGCCGCCAAACTCTGTC-3'	-5.65
6	Kinectin 1 (Kinesin receptor)	F: 5'-TTCCCCAGAAACGGAGTCTTC-3' R: 5'-TGAGCTGTTGGTTTACCGCC-3'	1.16
7	Cardiomyopathy associated 1 (CMYA1)	F:5'-CACTGCCCCAGGACTGAAGT-3' R:5'-AAAGGGAAATGGCCCACAGTA-3'	-25
8	Insulin-like growth factor binding protein 5 (IGFBP5)	F: 5'-TGTACCTGCCCAATTGTGACC-3' R: 5'-CGTACTTGTCCACGCACCAG-3'	1.56
9	Myosin, heavy polypeptide 3, skeletal muscle, embryonic (MYH3)	F: 5'-ACGGTGAAGGACCTGCAGC-3' R: 5'-CAGCTCTCGGATCCTGGTCTC-3'	-8.77
10	Myosin, heavy polypeptide 7, cardiac muscle, beta (MYH7)	F: 5'-CAGAAGCGCAACGCAGAGT-3' R: 5'-CGCAGCAGGTTTTTCCTGTC-3'	-25
11	Myosin, light polypeptide 2, regulatory, cardiac, slow (MYL2)	F:5'-GAAACTTAAGGGAGCGGACCC-3' R:5'-GCATTTCCCGAACGTAATCAG-3'	-20.41
12	Nedd4 family interacting protein 1 (NDFIP1)	F: 5'-CCTGACCACTTCAGCTGCAG-3' R:5'-CAGGGAAATAGGTGGAAAACCTG-3'	2.15
13	Ribosomal protein S3A (RPS3A)	F: 5'-CCTGACCACTTCAGCTGCAG-3' R:5'-CAGGGAAATAGGTGGAAAACCTG-3'	-3.78
14	Titin (TTN), transcript variant novex-2	F: 5'-CAAAAATTTCCGTGGCCAGT-3' R:5'-GTGTCACCACTTGTTCTCAATACTACC-3'	-43.48
15	glyceraldehyde-3-phosphate dehydrogenase, G3PDH	F: 5'-CAACTGCTTAGCACCCCTGG-3' R: 5'-CAGTCTTCTGGGTGGCAGTGA-3'	control

### RT-PCR detection

The primers were designed according to the sequences of the six new ESTs. The sense primer for A1 was 5'-CATTGAGGGAGCATGTTTAG-3' and the antisense primer was 5'-AGAATATGCAACCAGAAGAG-3'; sense primer for 5A-C was 5'-GACACAACCTGACAAATGGGATAA-3' and the antisense primer was 5'-CCTGTTAGCCATTTATATATCGTC-3'; the sense primer for 2A-A was 5'-GAGAAAAATAGAGATCTAAAGAGGG-3', the antisense primer for 2A-A was 5'-GCATCTGTGACAACATAGTCCTGAC-3'; the sense primer for 7B-B was 5'-GATACAGGCCAGTGTAGAATTATG-3', the antisense primer was 5'-GACACCAAATGTCCTGAA CAATCA-3'; the sense primer for 11A-A was 5'-ATGTTCAGCAACCAGGGAGTCTGTA-3', the antisense primer was 5'-AAAGCAGCAGAGTGCAGAGAGACAG-3'; the sense primer for 24A-A was 5'-GCTTGATGGGGACATAACCGATAGC-3', the antisense primer was 5'-CATTGAAGGAGAATACCCAAGTATGC-3'. One μg of total RNA was used to synthesize the first strand of cDNA using superscript II (Gibco). The PCR amplification was carried out in 25 μl of reaction volume containing 0.5 μl of reverse transcriptional products of total RNA from various tissues as the template, 1×PCR buffer, 1.5 mM MgCl2, 200 mM dNTPs, 0.5 μM primer, and 1 unit of Taq polymerase. After a 4 min denaturation at 94°C, PCR was performed for 28 cycles. Each cycle consisted of 94°C for 30 s, 60°C for 30 s and 72°C for 1 min, followed by a 72°C elongation for 6 min. Five μl of each PCR product was electrophoresised on a 1% agarose gel. GAPDH was amplified as the control with the same templates. The upstream/downstream primers of GAPDH were 5'-GACCACAGTCCATGCCATC-3'/5'-ACCAGG AAATGAGCTTGACA-3'.

### Histochemistry and immunohistochemistry

Cryosections, 10 μm thick, were stained with hematoxylin and eosin (H & E) according to routine protocol. Dystrophin and Emerin were analyzed by immunohistochemistry with the goat polyclonal antibody against the C-terminals of dystrophin (Santa Cruz) and Emerin (Santa Cruz), respectively, and endogenous peroxidase activity was blocked with 3% H_2_O_2 _for 15 min. Sections were incubated in primary antibodies (1:100) overnight at 4°C, then incubated with anti-goat IgG-HRP (1:100, Santa Cruz) for 1 hour, followed by staining with diaminobenzidine for 5 min at room temperature. Lastly the sections were counterstained with hematoxylin.

### Antibodies and Western blot

Antibodies against Dysferlin (E20), α-Sarcoglycan (D-20), β-Sarcoglycan (A-17), γ-Sarcoglycan (D-18), α-Tubulin, and horseradish peroxidase (HRP)-conjugated second antibodies were from Santa Cruz Biotechnology. For protein extraction from tissues, 100 mg of tissue was rapidly homogenized in 0.5 mL of homogenization buffer [50 mmol/L Tris-HCl (pH 7.5), 150 mmol/L NaCl, 0.5% NP40, 50 mmol/L NaF, 1 mmol/L Na3VO4, 5 mmol/L h-glycerophosphate, m1 mmol/L DTT, 1 mmol/L phenylmethylsulfonyl fluoride] and the lysate was clarified by centrifugation at 14,000 × g for 20 minutes. Boiled samples with 2 × SDS loading buffer were loaded onto polyacrylamide gel (8% for α-, β-, γ-sarcoglycan, 7% for dysferlin detection). After electrophoresis, the proteins were transferred onto polyvinylidene difluoride membrane (PALL). The protein blots were blocked with 10% milk in TBST buffer for 1 hour, and then incubated for another 1 hour at room temperature with primary antibody (1:1000 dilutions with 5% milk). The secondary antibody used in the immunoblot was a 1:2,000 dilution of HRP-linked anti-immunoglobulin G (IgG). The enhanced chemiluminescence reagent (Amersham Biosciences) was used as the substrate for detection and the membrane was exposed to an X-ray film for visualization.

## Results

### Clinical data about the patients from a consanguineous Chinese AR-LGMD family

**Figure 1:**
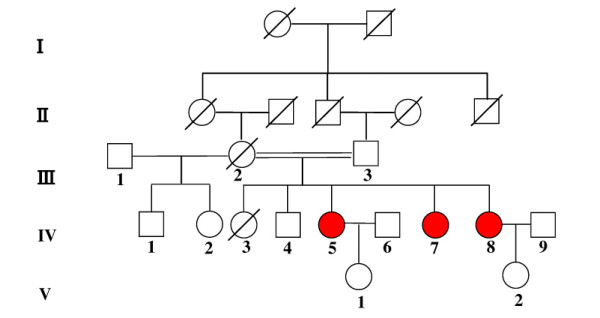
A consanguineous Chinese AR-LGMD family with three affected sisters.

Here we report a consanguineous Chinese AR-LGMD family with three affected sisters (fig. 1). The patients' mother (III-2) married two times and the children born from her first marriage were all normal. However, her second marriage was to her cousin and three daughters (IV-5, IV-7, IV-8) of five children suffered muscular weakness and dystrophy. The patients' mother (III-2) died of a stroke at the age of 61. The children from two of the patients are healthy at ages twelve and five, respectively (V-1, V-2). The consanguineous marriage and the horizontal pedigree pattern suggest that they suffer from a recessive genetic disease and that their unaffected parents are heterozygous carriers.

**Figure 2:**
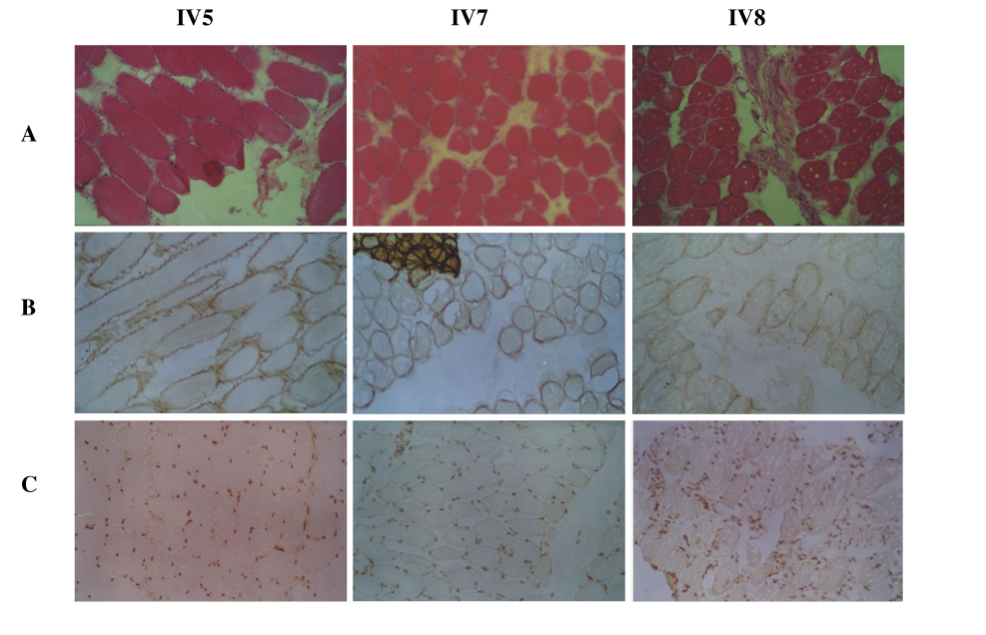
Histochemical and immunohistochemical staining in muscle biopsies from AR-LGMD patients. Hematoxylin and eosin (H&E) staining (panel A), dystrophin expression (panel B), and emerin detection (panel C).

The affected three sisters did not complain of muscle weakness until adolescence. The clinical data are summarized in table 1. The onset occurred during the late second decade of their lives. All patients had muscle weakness, predominantly in the pelvic and shoulder girdle musculatures, and were not ambulant by themselves at the time of the muscle biopsy. Two of them also showed mild hypertrophy of the gastrocnemius muscles. The serum CK levels of three patients were 2113.0 U/L, 2203.0 U/L, and 3000.0 U/L, respectively, which were approximately ten times higher than the normal SCK level (24~200 U/L). Electromyography (EMG) indicated that the pathological changes were due to myopathic rather than neurogenic damage. The morphological alterations of muscle biopsy (quadriceps femoris) observed by HE staining showed the typical dystrophic changes (Fig. 2A). None of patients had cardiac failure, involvement of the respiratory, oculopharyngeal or gastrocnemius muscle atrophy. In addition, the possibility of the patients suffering from BMD/DMD or EDMD was ruled out by immunohistochemical analyses of dystrophin and emerin proteins, which were detected on the sarcolema and nuclear membranes in the patients' samples (Fig. 2B,2C). Taken together, our data suggest that the three patients from this family suffer the autosomal recessive LGMD (AR-LGMD).

**Table 1:** Summary of the clinical data of three patients

Physical and clinical examination	Clinical symptoms or indications
Physical development	Normal
Psychological development	Normal
Age onset	Two sisters in the second decade, the oldest in the third decade
First symptoms	Difficulty in climbing stairs and standing up from chair
Pattern of muscle involvement	Lower and upper limb-girdle muscle
Clinical course	Slow
Pseudohypertrophy of calf	Transient calf hypertrophy at early stage
cardiomyopathy	No
Serum creatine kinase (SCK)	Very high, approximately 10 times than normal
EMG	Myopathic damage
Muscle biopsy	Typical muscle dystrophic change

### Molecular diagnosis of AR-LGMD patient

**Figure 3:**
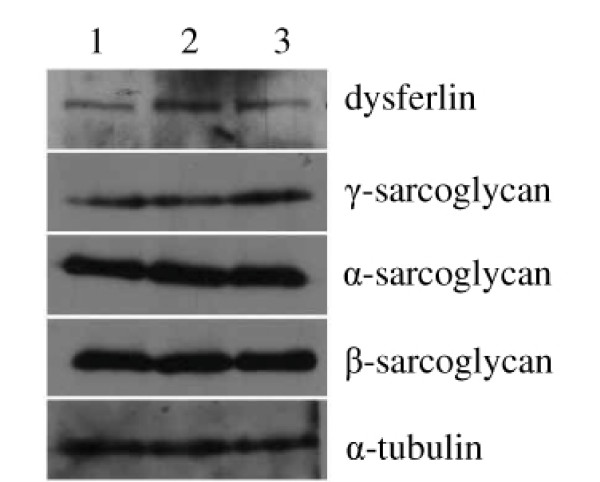
The expression analyses of α-, β-, γ-sarcoglycan and dysferlin protein in the muscles of patient by Western blot. The total proteins extracted from skeletal muscles of normal person (lane 1), relative normal muscle biopsies of patient IV-7 (lane 2), and dystrophic muscle biopsies of patient IV-7 (lane 3).

There are at least ten subtypes of AR-LGMD, named LGMD2A to LGMD2J. Genes involved in LGMD2A-2J are calpain 3, dysferlin, γ-, α-, β-, δ-sarcoglycans, telethonin, trim32, fukutin related protein, and titin. We sequenced two genes associated with AR-LGMD (calpain3 and telethonin), but no mutations of those two genes were detected in the patients, thus excluding LGMD2A and LGMD2G. Then we detected the expressions of γ-, α-, β-sarcoglycan and dysferlin proteins in the muscles of the patient (IV-7) by Western blot. The results (Fig. 3) showed the normal expressions of γ-, α-, β-sarcoglycan and dysferlin proteins in the muscles of the patient (IV-7), thus LGMD2B-LGMD2E should be excluded.

### Differential gene expression pattern between the relative normal and the dystrophic muscle from the same LGMD patient

**Figure 4:**
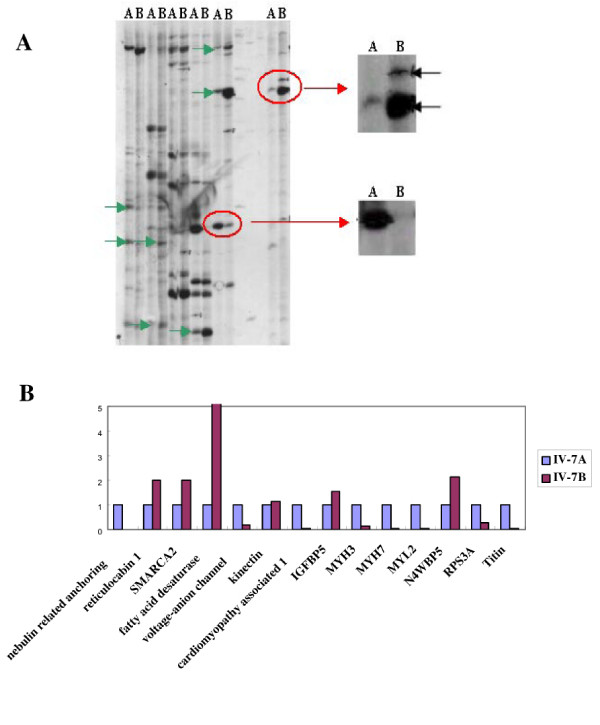
A typical sequencing gel (panel A, the arrows indicate the differentially expressed bands), real time qRT-PCR validation (panel B). The relative normal muscle (A) and the dystrophic muscle (B).

For identifying additional genes involved in the pathogenesis of AR-LGMD, the DD-RT-PCR was performed with RNAs isolated from relative normal (deltoid) and dystrophic skeletal muscle biopsies (quadriceps femoris) from the same patient (IV-7). Three anchored primers in combination with ten arbitrary primers (thirty primer pairs in total) were used in our experiments. Results from Fig. 4A showed an example of the differentially displayed polyacrylamide gels, and sixty differentially expressed bands were recovered from these gels for further sequence analysis. Among these sequenced cDNA fragments, there were 31 known genes, whereas the other 15 ESTs belonged to 12 unknown genes (table 2). According to the gene functions, the differentially expressed genes obtained from our DD-PCR were classified into several groups, including muscle developmental and structural proteins, ion channels, energy metabolisms, signal transduction and regulators (table 2).

**Table 2:** Differentially expressed genes between relative normal and dystrophic muscles from the same AR-LGMD patient

Accession no.	Gene title (gene symbol)	Gene expression pattern in dystrophic muscle
***Muscle development and function***		
NM_133437	Titin (TTN), transcript variant novex-2	Down
NM_002470	Myosin, heavy polypeptide 3, skeletal muscle, embryonic (MYH3)	Down
NM_000257	Myosin, heavy polypeptide 7, cardiac muscle, beta (MYH7)	Down
NM_000432	Myosin, light polypeptide 2, regulatory, cardiac, slow (MYL2)	Down
NM_194293	Cardiomyopathy associated 1 (CMYA1)	Down
NM_004543	Nebulin	Down
NM_006175	Nebulin-related anchoring protein (NRAP), transcript variant 1	Down
***Ion homeostasis***		
NM_003374	Voltage-dependent anion channel 1	Down
***Energy metabolism***		
NM_002612	Pyruvate dehydrogenase kinase, isoenzyme 4 (PDK4)	Down
NC_001807	NADH dehydrogenase subunit 2	Down
NM_013402	Fatty acid desaturase 1 (FADS1)	Up
***Signal transduction and regulator***		
NM_182926	Kinectin 1 (Kinesin receptor)	Up
NM_002901	Reticulocalbin1, EF hand calcium binding domain	Up
NM_014365	Heat shock 22 kDa protein 8 (HSPB8)	Down
NM_206876	Protein phosphatase 1, catalytic subunit, beta isoform	Down
NM_030937	Cyclin L2 (CCNL2)	Down
NM_014001	ADP-ribosylation factor binding protein GGA3	Down
NM_003566	Early endosome antigen 1	Up
NM_030571	Nedd4 family interacting protein 1 (NDFIP1)	Up
NM_000599	Insulin-like growth factor binding protein 5 (IGFBP5)	Up
NM_003916	Adaptor-related protein complex 1, sigma 2 subunit	Down
***Regulator for gene expression and protein translation***		
NM_139045	SWI/SNF related, matrix associated, actin dependent regulator of chromatin, subfamily a, member 2 (SMARCA2), transcript variant 2	Up
NM_012433	Splicing factor 3b, subunit 1, 155 kDa (SF3B1)	Up
NM_014752	Signal peptidase complex subunit 2 homolog (S. cerevisiae) (SPCS2), mRNA	Down
NM_001006	Ribosomal protein S3A (RPS3A)	Down
***Others***		
NM_020384	CLDN2 gene for claudin 2	Down
NM_016056	Transmembrane BAX inhibitor motif containing 4	Down
AF168681	CRIM1 protein gene, partial cds; and FEZ2 gene	Up
NM_001017977	IQ motif and WD repeats 1 (IQWD1), transcript variant 2	Down
BK001418	Metastasis associated in lung adenocarcinoma transcript 1 long isoform, transcribed non-coding RNA	Up
AF268386	DPDP-3 dental pulp-derived protein 3	Down
***Unknown genes***		
DW009597	3A-B	Down
EC268059	5A-C	Up
EC268060	24A-A	Down
EC268061	5A-D	Down
EC268062	7B-B	Down
EC268063	2A-A	Down
EC268064	18B-C	Up
EC268065	29B-A	Up
EC268066	25A-A	Down
EC268067	23A-C	Down
EC268068	29A-C	Down
EC268069	A1	Up

The differential expression of 14 genes was confirmed by real time qRT-PCR (table 3 and Fig. 4B). Our data demonstrated that the genes encoding structural proteins of skeletal muscle fibers (titin, myosin heavy and light chains, and nebulin) were dramatically downregulated in dystrophic muscles compared to the relative normal muscles, which was consistent with previously published data [9]. These results further confirmed that the integrity of skeletal muscles consisting of those cellular structural proteins play very important roles in maintaining the physiological functions of the skeletal muscles. However, these results were different from previously reported gene expression profiling in DMD patients[10,11], suggesting that different molecular mechanisms are most likely involved in the pathogenesis of this unique type of muscular dystrophy. Interestingly, we have provided experimental evidence to show that the genes encoding the proteins involved in signal transduction and gene expression regulation [reticulocalbin 1, kinectin 1, fatty acid desaturase 1, IGFBP5, Nedd4 family interacting protein 1 (NDFIP1), and SMARCA2 (SWI/SNF related, matrix associated, actin dependent regulator of chromatin, subfamily a, member 2)] were up-regulated in the dystrophic muscles. These results suggest that the pathogenesis development of LGMD is a complicated process and further analysis of the function of these genes in the pathogenesis of LGMD might provide valuable mechanistic information for understanding the molecular mechanism of LGMD development. Additionally, the functional investigation of those differentially expressed unknown genes could offer new information for revealing the pathogenesis of LGMD.

### Tissue specific expression patterns of several differentially expressed unknown genes

**Figure 5:**
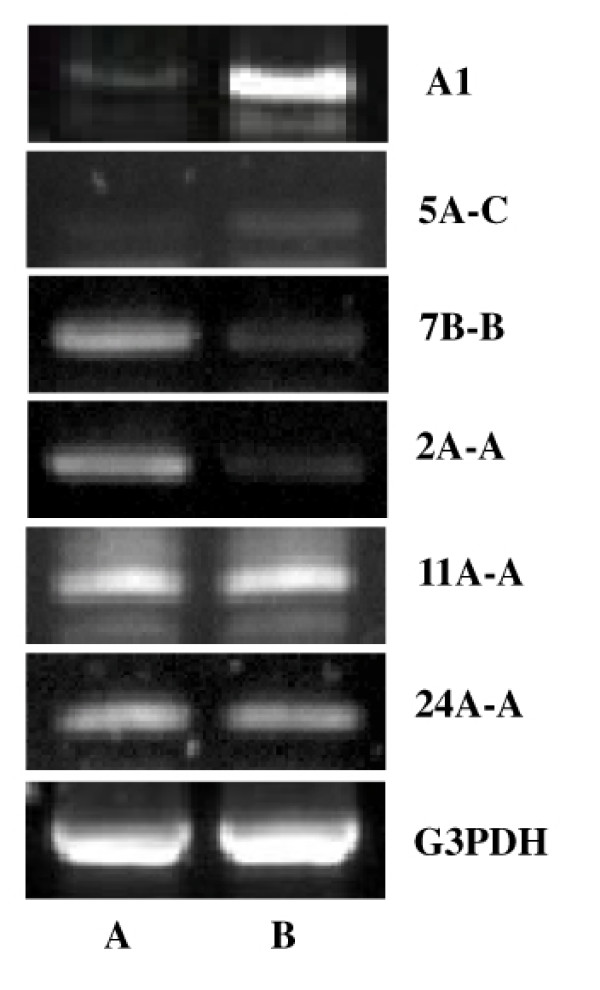
Confirmation of the differential expression of several unknown ESTs between the relative normal and dystrophic muscle tissues by semi-quantitative RT-PCR. The relative normal muscle (A) and the dystrophic muscle (B).

The semi-quantitative RT-PCR method was used to validate the differential expression of these unknown genes between the relative normal and dystrophic tissues. Total RNAs were isolated from the relative normal and pathological skeletal muscle tissues, and the amount of mRNA was normalized equally by the internal control, G3PDH. The primers were designed specifically for 6 new ESTs (A1, 5A-C, 11A-A, 7B-B, 2A-A, 24A-A) as indicated in the Materials and methods. The results indicated that the expression of A1 and 5A-C were up-regulated while 7B-B and 2A-A were down-regulated in the dystrophic skeletal muscle. There were no obvious expression changes of the ESTs 11A-A and 24A-A observed (fig. 5).

**Figure 6:**
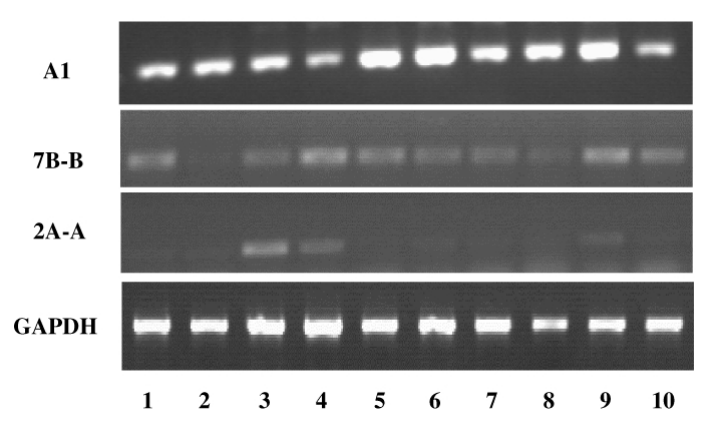
The tissue specific expression of the three unknown ESTs was analyzed by RT-PCR. Lane 1~10 are testes, liver, heart, quadriceps, intestine, kidney, spleen, pancreas, lung and brain, respectively. G3PDH expression was also detected and used for sample normalization.

For more functional information of these three differentially expressed unknown genes, the tissue distributions of the mRNAs were determined by RT-PCR. Total RNAs were isolated from various tissues of normal people, including the testes, liver, heart, quadriceps, intestine, kidney, spleen, pancreas, lung, and brain. The results indicated that 2A-A was mainly expressed in cardiac and skeletal muscles (Fig. 6), and as shown in Fig 5, 2A-A was downregulated in the dystrophic skeletal muscle, these findings suggest that 2A-A may play important roles in skeletal muscle development and the pathogenesis of LGMD. The mRNA of A1 and 7B-B was detected in all the tested tissues (Fig. 6). Interestingly, although the expression level of A1 was low in the skeletal muscle of the normal person, it was up-regulated greatly in pathological skeletal muscles (fig. 5), indicating that overexpression of A1 may have a functional role in the pathogenesis of muscular dystrophy.

## Discussion

Limb-girdle muscular dystrophy (LGMD), a kind of progressive muscular dystrophy (PMD), is a group of heterogeneous disorders with variable clinical and genetic features including autosomal dominant and recessive subgroups [12], in which the pelvic and shoulder girdle musculatures are predominantly or primarily involved. This kind of disease is also characterized by increased SCK levels, muscle fiber necrosis, and regeneration [13]. LGMD varies greatly from patient to patient in the age of onset, in disease progression and in the distribution of the affected muscles [14]. At least 13 genes, 3 autosomal dominant and 10 autosomal recessive, responsible for LGMDs have been identified [7,8]. Genotype-phenotype associations have been studied for the different types of LGMD in an attempt to promote our comprehension of its underlying pathological mechanisms. However, from the published information about the functions of those 13 genes, it is difficult to interpret the pathogenesis of the LGMDs' complex phenotype. Therefore, searching for the LGMD related genes and investigating their functions associated with disease development is still necessary, not only for a better understanding of the pathogenesis of LGMDs but also for the molecular diagnosis of LGMDs in a clinical setting.

In this study, we reported a Chinese family with three sisters suffering from AR-LGMD. In order to identify the genes associated with the pathogenesis of AR-LGMD, DD-RT-PCR was used to compare the gene expression differences between the relative normal and dystrophic skeletal muscles from the same LGMD patient, thereby search for candidate genes involved in the pathogenesis of AR-LGMD. We have found that 31 known genes and 12 new ESTs were differentially expressed between the normal and dystrophic tissues and that the differentially expressed known genes include structural proteins, anion channels, enzymes and signal transduction molecules.

From our experimental data, the expression levels of many genes, which encode for structural proteins of skeletal muscle fiber (such as MYH3, MYH7, MYL2, titin, nebulin related anchoring protein, and voltage-anion channel), were dramatically decreased in the dystrophic muscles compared to the relative normal tissues. Myosins are a large family of protein motors that contain at least 18 different classes [15]. They interact with actin filaments to generate a broad spectrum of eukaryotic cell movements that include phagocytosis, vesicle transport, cytokinesis and maintenance of cell shape in addition to their well-known role in muscle contraction [16,17]. Titin is a giant filamentous protein that forms a separate myofilament system in both skeletal and cardiac muscles. Titin has its NH2 terminus embedded in the Z-band and extends with its COOH terminus localized in the middle (M-band) of the aligned myosin filaments (A-band) [18,19]. The interaction of myosin, actin and titin play important roles in muscle contraction. Therefore, the expression alterations of these genes could directly affect the development and pathological changes of skeletal muscles, thus being implicated in the pathogenesis of muscular dystrophy.

We also found some differentially expressed transcripts encoding protein enzymes involved in protein synthesis and modification, energy metabolism, and gene expression regulation. The gene expression of the catalytic subunit (beta isoform) of PP1 was down-regulated in dystrophic muscles compared to the relative normal muscle. Protein phosphatase-1 (PP1) is 1 of 4 major serine/threonine-specific protein phosphatases involved in the dephosphorylation of a variety of proteins. Therefore PP1 plays a key role in numerous biological processes such as glycogen metabolism, cell cycle regulation, smooth muscle contractions, and protein synthesis [20,21]. We found that the expression level of voltage-dependent anion channels (VDAC) was decreased in dystrophic muscle compared to the relative normal muscle. VDACs are abundant 30-kDa mitochondrial outer membrane proteins found in all eukaryotes serving as a binding site for cytosolic hexokinase, providing the enzyme with preferential access to the ATP derived from oxidative phosphorylation [22,23]. Energy metabolism and skeletal muscle contraction cannot be carried out without VDACs. Although the expression alteration of these genes could have a close relationship with LGMD disease, their functional roles in the pathogenesis of LGMD need to be further investigated.

The altered expression of 6 new ESTs in the dystrophic muscles has been confirmed by the semi-quantitative RT-PCR method, and their tissue specific expression patterns were analyzed by RT-PCR. The results showed that the mRNA level of two ESTs (A1 and 5A-C) were up regulated in the dystrophic skeletal muscle, whereas two of them (7B-B and 2A-A) were down regulated. The tissue distribution and altered expression pattern of these ESTs provide important clues for understanding their function in the skeletal muscle development and pathogenesis of LGMDs. Although the expression level of A1 is very low in the skeletal muscle of the normal person, it is up-regulated in pathological skeletal muscles. Thus, the changes in A1 expression have a close relationship with the pathogenesis of muscular dystrophy. Abundant expression of 2A-A mRNA was mainly detected in cardiac and skeletal muscles. It might also be of some interest to further investigate the role of transcripts whose functions are still unknown.

## Conclusion

In summary, the functional analysis of these expression-altered genes in the pathogenesis of LGMD could provide additional information for understanding possible molecular mechanisms of LGMD development.

## Competing interests

The author(s) declare that they have no competing interests.

## Authors' contributions

YZ carried out the cloning and confirmation of differentially expressed genes and drafted the manuscript. JY carried out the tissue expression pattern analysis of unknown ESTs. DC carried out immunohistochemistry. XZ carried out the DD-RT-PCR and participated in the manuscript preparation. XX carried out the immunoassays and clinical observations of patients. ST participated in the clinical observations of patients and the sequence alignment of differentially expressed genes. WY participated in the design of the study and the sequence analysis of calpain3 and telethonin. DZ conceived of the study, and participated in its design and coordination and helped to draft the manuscript. All authors read and approved the final manuscript.
